# Facing depression: evaluating the efficacy of the EmpkinS-EKSpression reappraisal training augmented with facial expressions – protocol of a randomized controlled trial

**DOI:** 10.1186/s12888-024-06361-3

**Published:** 2024-12-12

**Authors:** Marie Keinert, Lena Schindler-Gmelch, Lydia Helene Rupp, Misha Sadeghi, Klara Capito, Malin Hager, Farnaz Rahimi, Robert Richer, Bernhard Egger, Bjoern M. Eskofier, Matthias Berking

**Affiliations:** 1 Department of Clinical Psychology and Psychotherapy, Friedrich-Alexander-Universität Erlangen-Nürnberg (FAU), Erlangen, Germany; 2 Machine Learning and Data Analytics Lab, Department Artificial Intelligence in Biomedical Engineering (AIBE), Friedrich-Alexander-Universität Erlangen-Nürnberg (FAU), Erlangen, Germany; 3 Chair of Visual Computing, Department of Computer Science, Friedrich-Alexander-Universität Erlangen-Nürnberg (FAU), Erlangen, Germany; 4 Translational Digital Health Group, Institute of AI for Health, Helmholtz Zentrum München - German Research Center for Environmental Health, 85764 Neuherberg, Germany; 5 Department of Clinical Psychology and Psychotherapy, Friedrich-Alexander-Universität Erlangen-Nürnberg (FAU), Nägelsbachstraße 25a, 91052 Erlangen, Germany

**Keywords:** Depression, Cognitive reappraisal, Facial expression, Embodiment, Smartphone-based intervention, Machine learning, Kinesthesia

## Abstract

**Background:**

Dysfunctional depressogenic cognitions are considered a key factor in the etiology and maintenance of depression. In cognitive behavioral therapy (CBT), the current gold-standard psychotherapeutic treatment for depression, cognitive restructuring techniques are employed to address dysfunctional cognitions. However, high drop-out and non-response rates suggest a need to boost the efficacy of CBT for depression. This might be achieved by enhancing the role of emotional and kinesthetic (i.e., body movement perception) features of interventions. Therefore, we aim to evaluate the efficacy of a cognitive restructuring task augmented with the performance of anti-depressive facial expressions in individuals with and without depression. Further, we aim to investigate to what extent kinesthetic markers are intrinsically associated with and, hence, allow for the detection of, depression.

**Methods:**

In a four-arm, parallel, single-blind, randomized controlled trial (RCT), we will randomize 128 individuals with depression and 128 matched controls without depression to one of four study conditions: (1) a cognitive reappraisal training (CR); (2) CR enhanced with instructions to display anti-depressive facial expressions (CR + AFE); (3) facial muscle training focusing on anti-depressive facial expressions (AFE); and (4) a sham control condition. One week after diagnostic assessment, a single intervention of 90–120-minute duration will be administered, with a subsequent follow-up two weeks later. Depressed mood will serve as primary outcome. Secondary outcomes will include current positive mood, symptoms of depression, current suicidality, dysfunctional attitudes, automatic thoughts, emotional state, kinesthesia (i.e., facial expression, facial muscle activity, body posture), psychophysiological measures (e.g., heart rate (variability), respiration rate (variability), verbal acoustics), as well as feasibility measures (i.e., treatment integrity, compliance, usability, acceptability). Outcomes will be analyzed with multiple methods, such as hierarchical and conventional linear models and machine learning.

**Discussion:**

If shown to be feasible and effective, the inclusion of kinesthesia into both psychotherapeutic diagnostics and interventions may be a pivotal step towards the more prompt, efficient, and targeted treatment of individuals with depression.

**Trial registration:**

The study was preregistered in the Open Science Framework on August 12, 2022 (https://osf.io/mswfg/) and retrospectively registered in the German Clinical Trials Register on November 25, 2024. Clinical Trial Number: DRKS00035577.

**Supplementary Information:**

The online version contains supplementary material available at 10.1186/s12888-024-06361-3.

## Background

Depression is a serious disease with symptoms such as persistent negative affect, anhedonia, and energy loss [1]. It is a common mental health problem with a global prevalence of 3.8% [2], a 50%-increased risk of mortality (for a meta-analysis, see [3]), and a high risk of chronicity [4]. Cognitive Behavioural Therapy (CBT) has proven effective in a vast number of studies [5], and is currently considered the gold-standard psychotherapeutic treatment for depression. However, consistently high attrition rates of up to 32% [6] and remission rates as low as 42% [7] suggest a dire need to further optimize the efficacy of CBT for depression. Moreover, since no more than 40% of individuals meeting criteria for depression receive minimally adequate evidence-based treatment [8], variations of CBT that can be easily disseminated are highly relevant for clinical practice.

**Fig. 1:**
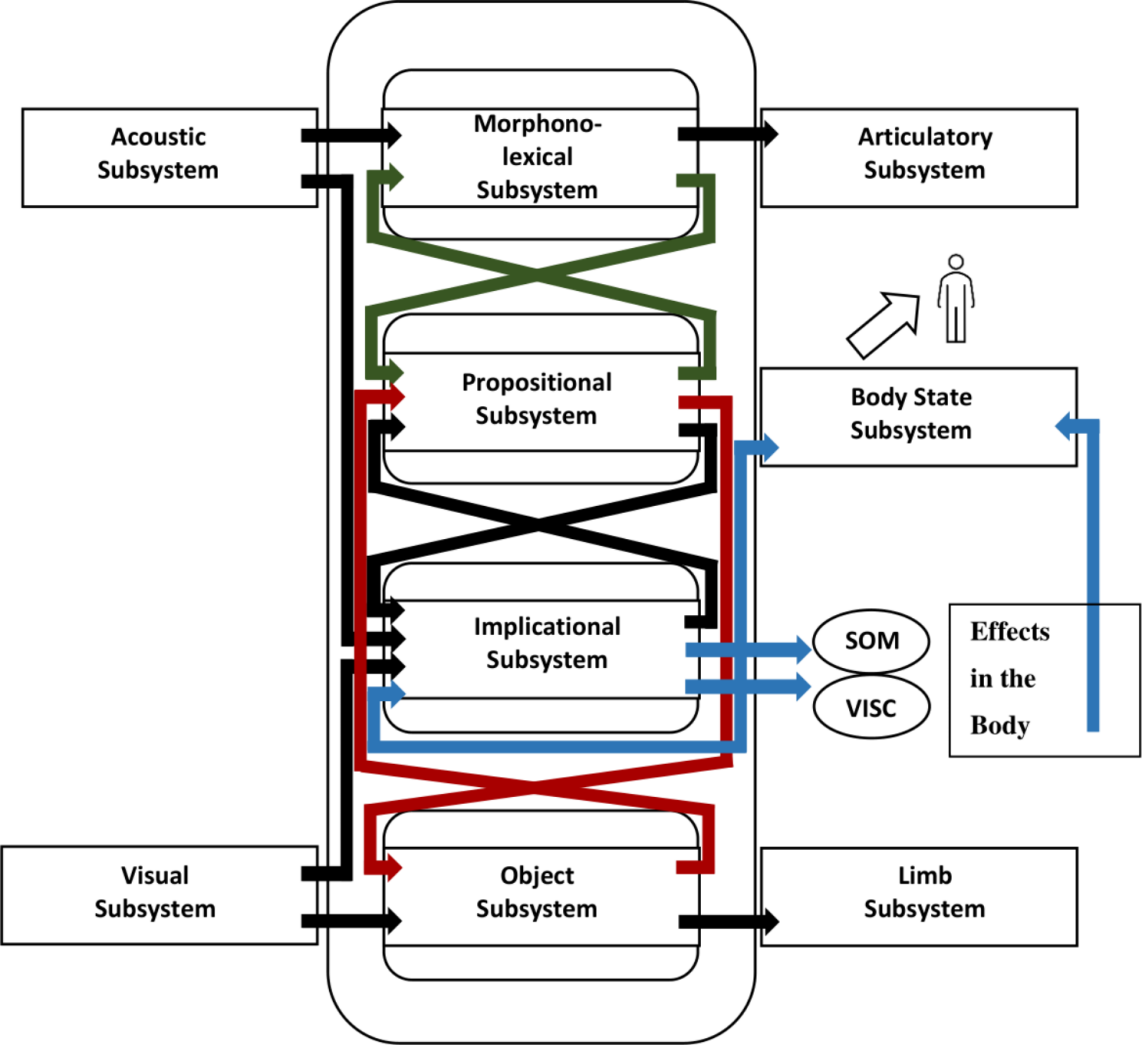
The generation of emotions in the Interacting Cognitive Subsystems Theory. Adapted from Affect, Cognition, and Change: Re-Modelling Depressive Thought (p. 59), by J. D. Teasdale and P. J. Barnard, 1993, Lawrence Erlbaum Associates. Copyright 1993 by Lawrence Erlbaum Associates Ltd. Adapted with permission

One of the primary techniques in depression-focused CBT is cognitive restructuring, which aims to systematically modify cognitive appraisal processes that trigger depressed mood [9]. In CBT, therapists work with their patients to identify automatic, depressogenic thoughts (e.g., “I am a complete failure”) and replace them with more functional ones (e.g., “I can do this”). To better consolidate the novel functional thoughts, patients are often coached to verbalize them repeatedly. It is commonly assumed that this eventually leads to a change in the underlying depressogenic beliefs and to the novel functional thoughts becoming integrated into the patient’s automatic thoughts. Consequently, there is a positive effect on the patient’s mood and on other symptoms of depression [10, 11]. However, patients often respond to invitations to verbalize anti-depressive thoughts with comments such as, “I can say that, but I cannot feel it” or “I can say that, but I do not believe it,” and they experience the exercise as futile and not worthy of practicing further. This clinical experience aligns with the theoretical assumptions of the Interacting Cognitive Subsystems Theory (ICS; [12]), which distinguishes *intellectual* and *emotional* beliefs. According to the ICS, cognitive restructuring exercises modify depressogenic thoughts on an intellectual level, but not necessarily their “felt meaning.” ICS explains this phenomenon with different subsystems that interact to generate emotions. The theory posits that depressogenic schemas are activated whenever any input information is appraised as highly aversive, uncontrollable, and stable over time. Self-verbalized (and thereby perceived) thoughts are only one possible input, in addition to input from the visual, acoustic, and body-state subsystem (see Fig. 1). Depressogenic thoughts such as, “I am a complete failure” are generated by the so-called *propositional subsystem*, which creates propositional meaning reflecting individual interpretations of situations (e.g., as aversive, uncontrollable, and stable) based on past experiences. The activity in this subsystem is purely cognitive, resulting in *intellectual beliefs*. In contrast, *emotional beliefs* require input from the so-called *implicational subsystem*. This subsystem processes thoughts transferred from the propositional subsystem, but also visual, auditory, and body-state-related information conveying an implicational level of meaning. The latter is a key strength of the ICS, as it highlights how cognitive-emotional processes may be moderated by physical input from the body. Hence, the effects of thoughts on emotions are considered to be in part moderated by “emotional proprioception” [13]. For example, if individuals without depression occasionally think “I am a complete failure,” the propositional meaning is likely to cue depressed mood. However, if the individual experiences such thoughts while standing upright, perceiving sufficient body power, and maybe even smiling to themself for underperforming so terribly that it is almost entertaining, this emotional proprioception has previously been associated with *successfully* meeting significant challenges. Hence, it carries the implicational meaning that the situation might not be as aversive, uncontrollable, and stable as suggested by the cued negative thought, and protect from the activation of depressogenic schemas. Contrastingly, in individuals with depression, the same negative thought is likely to coincide with slumped body posture, frowning, and sagging corners of the mouth. Since this proprioception has repeatedly been associated with situations that were indeed highly aversive, uncontrollable, and stable over time, they validate the automatic thought. As a result, the implicational subsystem triggers the depressogenic schema, which in turn cues depressed mood. Regarding the development and maintenance of clinical depression, ICS claims that the depressogenic affect then likely prompts further depressogenic interpretations in the propositional subsystem while inducing bodily changes such as slumped body posture, frowning, sagged corners of the mouth, etc. Thus, the negatively biased interpretation and the bodily changes reinforce each other and trigger the depressogenic schema. This interaction between (a) the depressogenic interpretations in the propositional subsystem and (b) depression-related changes in body-state is called the *cognitive interlock of depression*.

Drawing on these theoretical assumptions, we deduce that targeting emotional proprioception in addition to depressogenic thoughts, and thus, the *emotional* as well as the *intellectual belief*, might indeed further optimize the efficacy of cognitive interventions for depression. More specifically, anti-depressive kinesthesia, i.e., the subfacet of proprioception encompassing the awareness of body movement, appears to be particularly relevant, as it is a conscious act that the individual can purposefully engage in. Numerous empirical studies already provide evidence for a relationship between kinesthesia and emotional experience in general, and for depression in particular. For example, depression has consistently been linked to characteristic changes in gait and posture (for reviews, see, e.g., [14, 15]). Moreover, the experimental manipulation of gait and posture has been shown to lead to changes in emotional experience and depression-related cognitive processing (e.g., [16–24]). That said, facial expression could be even more critical in the context of inducing emotional beliefs since facial muscles are very sensitive and quick to respond to emotional experiences [25]. Indeed, the relationship between emotional experience and facial expression has been proven in multiple studies (for meta-analytic evidence, see [26]). More specifically, depression has been shown to be associated with characteristic facial expressions (e.g., [27, 28]) and, particularly, less facial expression of positive affect (as reviewed by [29]). In studies using electromyography (EMG) measures, individuals with depression display greater overall activity of the musculus corrugator supercilii (the so-called “grief muscle”) when confronted with affective pictures [30, 31] and during negative thinking [32]. Moreover, one study found an association between activation in the musculus corrugator supercilii and clinical improvement [33]. Consistently, induction of a sad facial expression or contraction of the musculus corrugator supercilii has been shown to be associated with a more negative interpretation of ambiguous stimuli (which is one of the cognitive biases found in depression) in a healthy sample [34]. Additionally, a recent meta-analysis of five randomized controlled trials (RCTs) showed that blocking the musculus corrugator supercilii via botulinum toxin injection can lead to a significant reduction of depressive symptoms in patients with depression compared to a placebo control group with a large effect size (*d* = 0.98; [35]).

Despite these encouraging findings, there are important gaps in the current literature. First, many studies focus on only one or a few kinesthetic parameter(s) (e.g., muscle activity), even though it can be assumed that the importance of the kinesthetic input is proportional to the number of kinesthetic parameters included in the analyses. Secondly, few studies focus on the interaction between kinesthesia and cognitive processes, although their interaction could be more important than each independent domain. Thirdly, current studies on treating depression by manipulating facial expressions use methods that are too invasive to be easily integrated into psychotherapeutic treatments (i.e., injection of botulinum toxin; [35]). Most importantly, no study has investigated the effects of augmenting cognitive interventions for depression via systematic kinesthetic manipulation, nor have researchers specifically studied the effects of experimentally manipulating the degree to which anti-depressive and depressogenic self-statements are deliberately accompanied by validating/invalidating kinesthesia. This is puzzling considering both clinical experience and theoretical frameworks (e.g., ICS Theory) clearly suggest this could be a promising approach to achieve more sustainable effects of cognitive interventions for depression.

In the present study, we aim to fill these gaps in the depression and CBT literature pertaining the role of kinesthetic input (facial expression) in the modification of depressive mood. The study, “EmpkinS-EKSpression” is part of the Collaborative Research Center (CRC) “Empatho-Kinaesthetic Sensor Technology – Sensor Techniques and Data Analysis Methods for Empatho-Kinaesthetic Modeling and Condition monitoring” (EmpkinS, CRC 1483, German Research Foundation). Its overarching goal is to derive information about clinically relevant states from minimally disturbing and non-invasive assessments of motion for research, diagnostic, and intervention purposes. Specifically, we intend to examine the extent to which the efficacy of an automated, smartphone-based cognitive reappraisal training (as a key cognitive restructuring technique) against depressed mood can be augmented by systematically pairing the vocalization of depressogenic self-statements with invalidating kinesthesia and the vocalization of anti-depressive self-statements with validating kinesthesia, respectively. We further aim to clarify the extent to which singular markers as well as the combined pattern of kinesthetic indicators are associated with depression and could, hence, be employed for the automated detection of depression.

## Methods

### Design

**Fig. 2:**
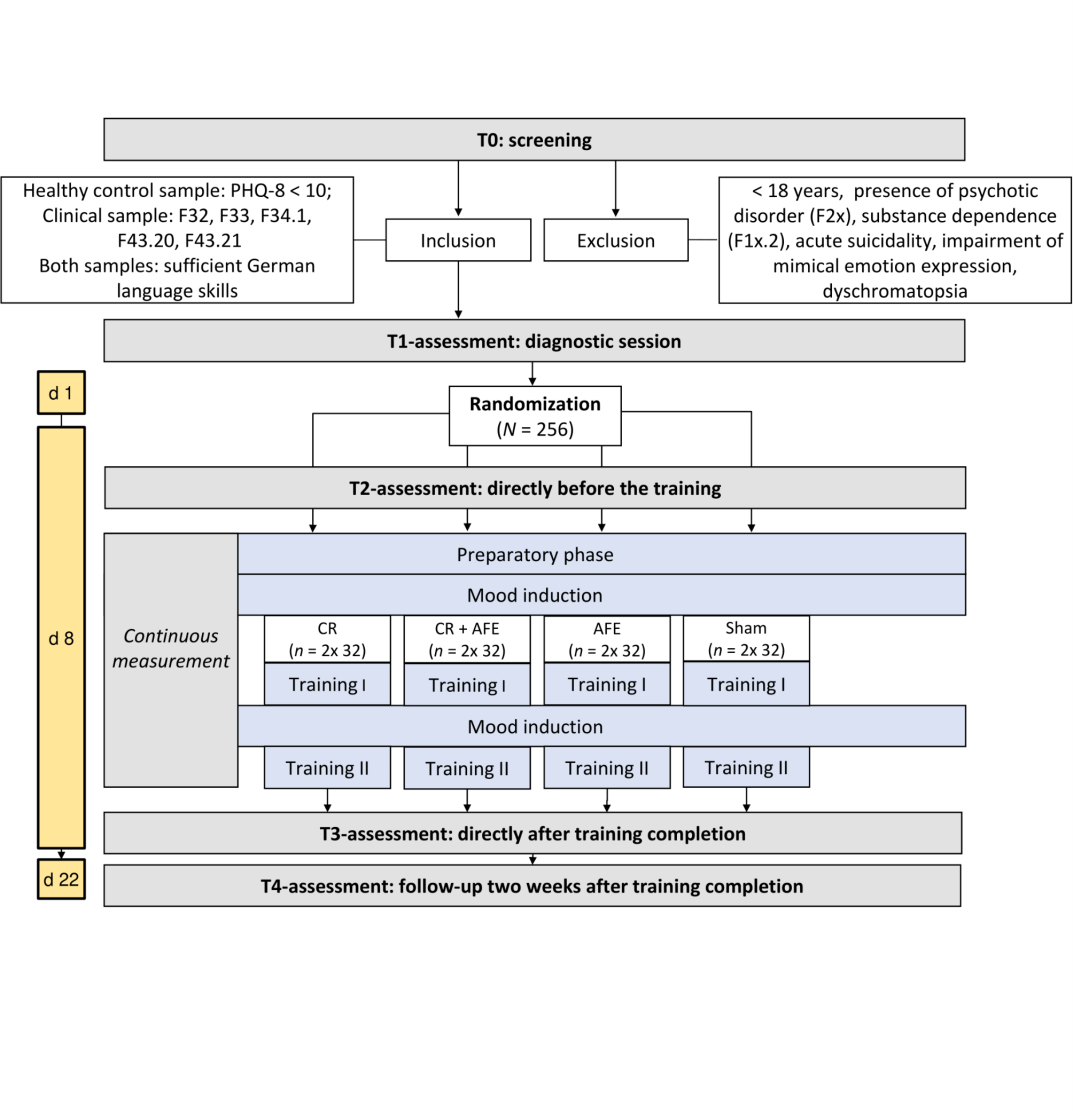
Study procedure. PHQ-8 = Patient Health Questionnaire-8; d = day; CR = cognitive reappraisal training; CR + AFE = cognitive reappraisal training enhanced by instructions to deliberately display anti-depressive facial expressions; AFE = facial muscle training focusing on anti-depressive facial expressions

To examine the relevance of kinesthesia for treatment and diagnosis of depression, we aim to conduct a four-armed, parallel, single-blind RCT (see Fig. 2) in accordance with the CONSORT statement [36]. The study is preregistered in the Open Science Framework (https://osf.io/mswfg/). It will be carried out at the EmpkinS Lab of the Friedrich-Alexander-Universität Erlangen-Nürnberg, structured around a smartphone-based training. There will be three intervention conditions: (1) a cognitive reappraisal training (CR), (2) CR enhanced by instructions to deliberately display anti-depressive facial expressions (CR + AFE), and (3) facial muscle training focusing on anti-depressive facial expressions (AFE). The control condition (4) will receive a sham intervention. Participants will be randomly allocated to one of the four study conditions with a 1:1 allocation ratio. A block randomization with a randomly varying block size of four or eight will be performed following a randomization list generated using the blockrand-package in R Studio [37]. To control for confounding expectation effects, participants will be blinded regarding the study condition they are allocated to, but will be debriefed after study completion. Moreover, to avoid biased assessments, researchers conducting the follow-up assessment will also be blinded to participants’ group allocation.

### Participants

We will recruit two participant groups of equal size with differing diagnostic status: one group of individuals with clinically relevant depressive symptomatology and a control group of individuals without depression. The diagnostic groups will be matched according to gender and age. For the depression group, the main inclusion criterion is the presence of a diagnosis from the depressive spectrum according to ICD-10 criteria (F32 ‘depressive episode’, F33 ‘recurrent depressive disorder’, F34.1 ‘dysthymia’, and F43.20 & F43.21 ‘adjustment disorder with depressive symptoms’ [38]) according to the Structured Clinical Interview for DSM-5 Disorders - Clinician version (SCID-5-CV; German Version: [39]). The main inclusion criterion for the group without depression is a score < 10 on the Patient Health Questionnaire (PHQ)-8 [40]. Additional inclusion criteria for both groups include being ≥ 18 years, being able and willing to provide informed consent regarding study participation, and having sufficient German language skills (at least level B2 according to the Common European Framework of Reference for Languages). Exclusion criteria included the presence of any psychotic disorder (ICD-10 F2x), any substance dependence disorder other than nicotine (ICD-10 F1x.2), acute suicidality, any impairment of facial expression (e.g., due to facial paralysis or recent Botox treatment or autism spectrum disorder), and any type of dyschromatopsia. To ensure maximum external validity, we refrain from applying further potential exclusion criteria such as other clinical comorbidities, or concurrent pharmaco- or psychotherapy, but will record this information.

A power analysis was conducted with G*Power [41] to determine the necessary sample size for a 2-group-comparison of each of the intervention conditions (CR, CR + AFE, AFE) with the sham control condition. In absence of similar studies serving as reference for the determination of a critical effect, a team of three clinical experts agreed that an effect size of *d* = 0.5 would be therapeutically relevant. The power analysis yielded a necessary sample size of *n* = 32 for each of the four intervention conditions and two diagnostic groups based on a 2-sided analysis, a critical alpha-level of α = 0.05, and a power of β = 0.80. Hence, we will include *N* = 256 participants in total, composed in equal parts of participants with depression (*n* = 128) and without depression (*n* = 128).

### Procedure

Recruitment will take place via newspaper and social media advertisements, mailing lists, and flyers on public bulletin boards. Furthermore, individuals with depression will be recruited via the outpatient clinic for psychotherapy of the Friedrich-Alexander-Universität Erlangen-Nürnberg, and by contacting psychiatric or psychosomatic clinics, counselling center, psychotherapists, and general practitioners in and around Erlangen via telephone or email.

The recruitment material will provide a QR code and link to the study website hosted by Unipark.com. Here, interested individuals will find extensive information on study aims and procedures (without jeopardizing blinding). Furthermore, they will be able to directly access the study screening questionnaire (T0) on demographic information, inclusion and exclusion criteria, and symptoms of depression. If eligible, participants will be invited to a first appointment in the laboratory for a diagnostic session (T1). During this session, after obtaining written informed consent, two diagnostic interviews (see Table 1) will be conducted by trained psychologists. All interviews will be video-recorded and 10% rated by a second expert to determine inter-rater reliability for diagnostic status. In case of ambiguity, the diagnostic status will be deliberated with experienced clinical psychologists. Moreover, participants will answer a set of questionnaires delivered on a computer tablet via Unipark.

**Table 1:** *Overview of measures per Time Point*

Variable	Instrument	Time point					
		T0	T1	T2	Continuous^a^	T3	T4
**Screening**							
Demographic information	self-developed	X					
Health-related information	self-developed	X					
Symptoms of depression	PHQ-8/-9	X	X				
**Diagnostic interviews**							
Clinical status	SCID-5-CV		X				
Symptoms of depression	GRID-HAMD		X				X
**Psychometric measures**							
*Primary outcome*							
Current depressed mood	self-developed		X		X		
*Secondary outcomes*							
Current positive mood	self-developed		X		X		
Symptoms of depression	CES-D			X			X
Current suicidality	self-developed		X	X		X	X
Emotional state	ERSQ-ES A		X				X
Dysfunctional attitudes	DAS-A		X				X
Automatic thoughts	ATQ-R		X				X
**Feasibility measures**							
Treatment integrity	RGB camera		X		X		
	self-developed		X		X		
Compliance	self-developed				X		
Usability	UEQ-S					X	
	self-developed					X	
Acceptability	UEQ-S					X	
	self-developed				X	X	
**Kinesthesia**							
Facial expression/body posture	RGB camera		X^b^		X		
	Azure Kinect depth camera				X		
	Smartphone camera				X		
**Further psychophysiological measures**							
Facial muscle activity^c^	Biopac MP160				X		
Respiration rate (variability)	Biopac MP160				X		
Heart rate (variability)	Biopac MP160				X		
	24 GHz & 61 GHz CW Radar systems (self-developed)				X		
Verbal acoustics	Microphone		X		X		
**Technical app data (e.g.**,** reaction time**,** item choice**,** credibility of emotion display)**	EmpkinS EKSpression app				X		

Note. T0 = Screening; T1 = diagnostic session one week before the training; T2 = assessment directly before the training; T3 = assessment directly after training completion; T4 = assessment two weeks after training completion. SCID-5-CV = Structured Clinical Interview for DSM-5 Disorders Clinician version; GRID-HAMD = GRID Hamilton Rating Scale for Depression; PHQ-8/-9 = Patient Health Questionnaire-8/-9; CES-D = Centre for Epidemiological Studies Depression Scale; DAS-A = Dysfunctional Attitude Scale version A; ATQ-R = Automatic Thoughts Questionnaire-Revised; ERSQ-ES A = part A of the Self-Report Instrument for the Assessment of Emotion-Specific Regulation Skills; UEQ-S = User Experience Questionnaire, short version

^a^Measures are taken continuously during the training. ^b^The main purpose of the video recordings during T1 was to determine inter-rater reliability of the diagnostic interviews. ^c^We will use a bipolar electromyogram to measure activity of the musculus corrugator supercilii, musculus zygomaticus major, musculus masseter (only in the AFE condition), and musculus orbicularis oculi

The training session will take place one week after the diagnostic session at the laboratory, taking approximately three hours in total. At the beginning, participants will be randomly allocated to study conditions. We will use numbered, sealed envelopes containing the respective allocation information. The envelopes will be prepared by a student assistant not otherwise involved in the study. Following a pre-assessment (T2) on a computer tablet via Unipark.com and the setup of the kinesthetic and physiological data acquisition (see below for details), participants will receive brief psychoeducational instructions about the interconnection of cognitions (thoughts), the body (e.g., facial expression) and emotion/mood. Then, they will complete the 90-120-minute intervention on a study smartphone. The training session will be concluded with a brief self-report post-assessment (T3).

Two weeks after the training session, a follow-up assessment (T4) will take place in the form of a telephone interview to reassess the severity of depressive symptoms using the Hamilton Rating Scale for Depression (HAMD). Additionally, participants will be asked to complete online questionnaires via Unipark.com, which they will receive via email one day before the telephone interview. Figure 2 outlines the study procedures. Participants will receive compensation in the amount of €40 up to €100 for individuals with depression in the final phase of the study, whereby psychology students will have the option to receive course credits instead of financial reimbursement. Further, individuals with any current psychiatric disorder according to the SCID-5-CV are offered priority on the waitlist of the outpatient clinic for psychotherapy of the Friedrich-Alexander-Universität Erlangen-Nürnberg.

All study sessions will be conducted by study therapists holding at least a bachelor’s degree in Psychology. They will be trained and supervised by clinical psychologists, and a trained and licensed CBT psychotherapist will be accessible by phone at all times. Moreover, all training procedures will be manualized to ensure best practice on the side of the study therapists.

### Smartphone-based intervention

In all conditions, the training will consist of five distinct phases in total (see Fig. 2): As an initial step, a preparatory phase will serve as a baseline and resting period for physiological measures. Then, a mood induction phase will be initiated to induce a depressed mood prior to the first training phase. After the first training phase, a second mood induction and a second training phase will follow. Preparatory phase and both mood induction phases will be identical for all participants. The two training phases will differ depending on the condition that each participant has been allocated to.

During the preparatory phase, different stimuli will be presented to the participant via smartphone. These will alternate between live self-recordings of the participant and verbal stimuli: “hopeless” and “happy” as words congruent or incongruent with depressiveness, respectively, and “me,” “the world,” and “the future” corresponding with Beck’s cognitive triad of depression [42]. Each stimulus will be presented for five seconds in randomized order following the display of a fixation cross (3 s.) and will be shown twice, adding up to a total of twelve stimulus presentations. Before the first and after each stimulus presentation, two one-item self-reports of the current level of depressed and positive mood, respectively, will be collected.

The subsequent mood induction will follow a validated procedure [43, 44] and consist of ten depressogenic cognitions shown on-screen (e.g., “*My future is absolutely hopeless*”), which the participant will read aloud while listening to music inducing depressed mood (i.e., excerpt from “Adagio in G minor” by Tomaso Giovanni Albinoni). After the mood induction, participants will again rate their current level of depressed and positive mood.

#### Training for the three intervention conditions

The two training phases will each start with detailed instructions by both the study therapist *and* on-screen via text and animations. In two subsequent practice runs, participants will then familiarize themselves with the respective exercises. Following these practice runs, each training phase will consist of 20 trials, appearing in a randomized order, accompanied by mood ratings. In all intervention conditions, participants will receive instructions and feedback with reinforcing statements, pictures, and animations in the smartphone app. Further, the study therapist will provide support and feedback throughout the training and, in the CR and CR + AFE conditions, rate the performance of the participants via the app. The app will be controlled by the study therapist with a computer tablet that will be connected to the app via a secure local network.

**Fig. 3:**
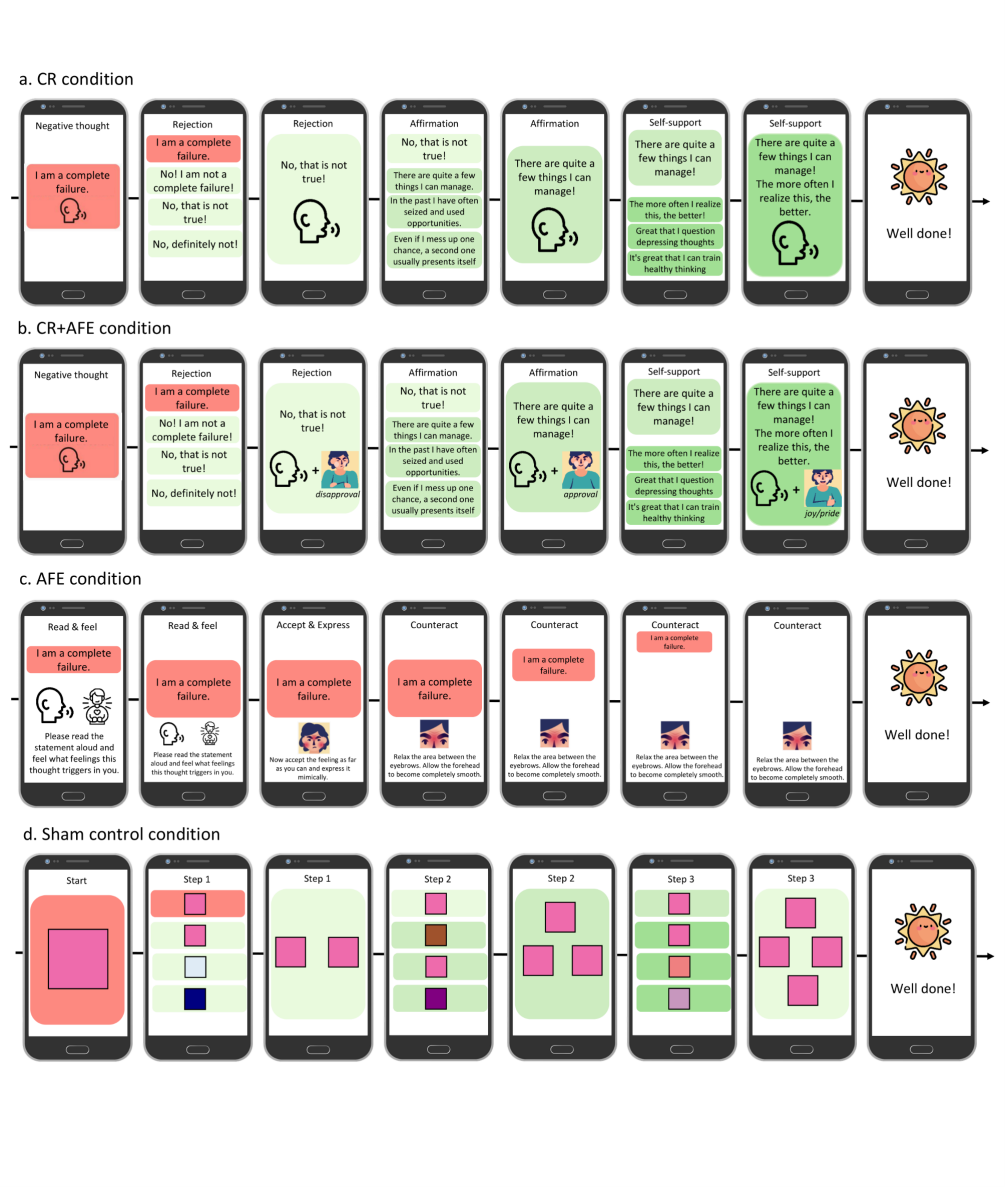
Trial flow of the four study conditions. CR = cognitive reappraisal training; CR + AFE = cognitive reappraisal training enhanced by instructions to deliberately display anti-depressive facial expressions; AFE = facial muscle training focusing on anti-depressive facial expressions

During the training, participants will be confronted with negative, depressogenic (e.g., ‘*I am a complete failure’)* and anti-depressive cognitions (e.g., ‘*I can do this’)* on the smartphone screen. Participants are instructed to read the statements aloud, briefly rate their mood, and counteract the depressogenic/strengthen the anti-depressive cognitions in different fashions, depending on the study condition (see Fig. 3). The sequence of the training phases will be balanced, so that half of the participants start with the depressogenic, and half with the anti-depressive cognitions. The cognitions used in the training will consist of items from the Beck Hopelessness Scale (German version: [45]), the Cognitive Triad Inventory (German version: [46]), the Automatic Thoughts Questionnaire (German version: [47]), and the Rosenberg Self Esteem Scale (German version: [48]) adapted and complemented by a team of three clinical psychologists with expertise in cognitive behavioral therapy for depression (including the senior author). Depending on the study condition, participants are instructed to deal with the statements in different fashions:

*CR*. To work with the cognitions, participants are instructed to respond to each of them in three steps. First, the screen will display three statements expressing *rejection* of the depressogenic cognition or *approval* of the anti-depressive cognition. Second, three statements *affirming* the rejection/approval, and third, three *self-supporting* statements will be presented on the screen. In each step, participants will choose the subjectively most fitting statement by selecting it on the screen and reading it aloud. To conclude the trial, participants again read aloud the affirming and self-supporting statement (depressogenic cognition training) or all three positive statements (anti-depressive cognition training) they have selected in a combined fashion.

*CR + AFE.* In the CR + AFE condition, participants will follow the same three steps as in the CR condition, except that they are instructed to mirror them with congruent kinesthesia including mime, posture, and gesture. Step 1 will be accompanied by rejection/approval, Step 2 by approval/confidence, and Step 3 by joy (trials 1 to 10) or pride (trials 11 to 20).

*AFE*. In the AFE condition, participants are instructed to first experience the elicited emotion, and then counteract depressogenic cognitions/strengthen anti-depressive cognitions by tensing or relaxing relevant facial muscles, for example, the forehead (i.e., the area between the eyebrows). In total, four different facial muscles are targeted in this condition: (1) m. corrugator supercilii or the “frowning muscle,” (2) m. zygomaticus, the muscle relevant to lifting the corners of the mouth to smile, (3) m. orbicularis oculi, a circular muscle around the eye activated when smiling, and (4) m. masseter or the “chewing muscle,” used when clenching one’s teeth, as well as different combinations of all four facial muscles. The exact number of combinations and repetitions can be found in Supplementary Table 1. When participants perform the respective expression, the study therapist commands the app to zoom-out depressogenic and zoom-in anti-depressive statements on the smartphone screen according to the intensity of participant’s performance as trainer-operated biofeedback.

#### Training for the sham control condition

For the sham control condition, the training consists of allocating geometric forms to a target object based on either their color (Training Phase 1) or their shape (Training Phase 2) in order to control for effects of interacting with a study therapist and a smartphone, as well as activation related to study participation.

### Measures

#### Demographic and health-related information, screening, and diagnostics

Demographic and health-related variables assessed include age, date of birth, German language skills, nationality, gender, height, weight, academic degree, professional occupation, current academic field, relationship status, lifetime diagnosis of a psychiatric disorder, as well as history of and current psychotherapy and medication treatment.

The PHQ-8 will be used to screen for current depressive symptomatology during online screening (T0). The 8-item self-report measure assesses the frequency of eight of the nine criteria for depression according to the DSM-5 [1] during the previous two weeks on a four-point Likert-scale (0 = *not at all* to 3 = *nearly every day*). In contrast to the PHQ-9 [49], the PHQ-8 does not assess suicidality or self-injury. To ensure provision of adequate psychological support when necessary, we exclusively assessed suicidality/self-injury while face-to-face with participants. Previous studies showed comparable validity of the PHQ-8 and PHQ-9 in assessing depression [40, 50]. The items of the PHQ-8 can be summed up to a total score with a maximum of 24. A score ≥ 10 indicates clinically relevant depression [40].

To assess the presence of psychiatric disorders according to DSM-5, we will conduct the SCID-5-CV (German version) as part of the diagnostic session (T1). It has demonstrated acceptable to excellent reliability [39]. For a more in-depth overview of the *severity of depressive symptoms*, the German version of the GRID-HAMD [51] will be used. The GRID-HAMD represents a revised version of the HAMD [52] and integrates a guideline for the semi-structured interview. Moreover, the GRID-HAMD assesses the frequency (4-point scale from *absent* to *almost all of the time*) as well as the intensity of symptoms (5-point scale from *absent* to *very severe*) during the previous week, allowing a dimensional rating of the severity of depression. In the current study, we will use the 21-item version of the GRID-HAMD complemented with the items, *helplessness*, *hopelessness*, and *worthlessness* of the HAMD-24. The GRID-HAMD has shown both validity, and acceptable to excellent reliability (Cronbach’s alpha = 0.78, ICC = 0.95; [51]). During the diagnostic session, the first part of the SCID-5-CV assessing current depressiveness (SCID-5-CV chapter A) and the HAMD will be combined to maintain a more pleasant conversation flow during the interview. While this requires a change in the order of HAMD-items, no changes to content will be made. The new item order of the combined tool can be found in Supplementary Table 2.

#### Primary outcome

*Current depressed mood* will be assessed using an eleven-point Likert scale from 0 to 10, with 0 = *no depressed mood at all* and 10 = *very strong depressed mood*, respectively. Participants will submit twelve mood ratings via smartphone during the preparatory phase, with one rating after each emotion induction, and one rating immediately before the first training item, as well as twice during each trial (once after the initial statement has been read out aloud and once after the last step of the trial has been completed). Thus, a total of 96 mood ratings for depressed mood will be compiled for each participant.

#### Secondary outcomes

Similarly to current depressed mood, *current positive mood* will be assessed by means of an eleven-point Likert-scale from 0 to 10, with 0 = *no positive mood at all* and 10 = *very strong positive mood*, respectively. The number of mood ratings for positive mood is the same as for depressed mood, adding up to a total of 96 positive mood ratings over the course of the training.

Beyond clinical interviews and single-item mood ratings, questionnaires will be administered at several points during the training. The German version of the Centre for Epidemiological Studies Depression Scale (CES-D; [53]) will be administered to assess self-reported symptoms of depression during the previous week. The 20 items are rated on a four-point Likert scale from 0 (*some of the time*) to 3 (*most of the time*). A total score can be obtained by summing up the items, with a score greater than 22 considered to be clinically relevant [53]. The CES-D has demonstrated good reliability and validity in several studies [54, 55].

*Current suicidality* will be assessed with a single, self-developed item on a six-point Likert scale from 1 (*no suicidality*) to 6 (*suicide attempt*).

*Emotional state* will be assessed with Part A of the Self-Report Instrument for the Assessment of Emotion-Specific Regulation Skills (ERSQ-ES; [56]). This scale measures feelings and mood during the previous week with 50 items on a 5-point Likert scale from 0 (*never*) to 4 (*all the time*). *Dysfunctional attitudes* will be assessed with the German short version of the Dysfunctional Attitude Scale version A (DAS-18-A; [57]). The short version consists of 18 items assessing respondents’ agreement with dysfunctional attitudes that are typically found among individuals with depression (e.g., “My value as a person depends on what others think of me”) during the last week on a 7-point Likert scale from 1 (*fully disagree*) to 7 (*fully agree*). The DAS-18-A has shown good validity and reliability in patients with depressive symptoms and in healthy individuals [57].

In addition, the 21-item German version of the Automatic Thoughts Questionnaire-Revised (ATQ-R; [47]) will be administered. The ATQ assesses the frequency of positive and negative automatic thoughts (e.g., “My life is a mess”) during the previous week on a five-point Likert scale from 1 (*not at all*) to 5 (*all the time*). It has shown acceptable to good reliability in the general population (Cronbach’s alpha = 0.67-0.86) and good reliability in a patient sample (Cronbach’s alpha = 0.84-0.94; [47]).

#### Feasibility measures

We will assess treatment integrity, compliance, usability, and acceptability to determine the feasibility of our intervention.

*Treatment integrity* is defined as study therapists’ adherence and competence (in accordance with [58]. A subsample (10%) of video-recorded training sessions will be used to rate treatment integrity. Adherence with the manual and therapist competence will be rated on a five-point Likert scale from 1 (*not at all*) to 5 (*very much*).

*Compliance* of participants in terms of commitment to the training will be rated by study therapists on a five-point Likert scale from 1 (*not at all*) to 5 (*very much*).

The *usability* of the intervention smartphone app will be operationalized with the short form of the User Experience Questionnaire (UEQ-S; [59]) and one self-generated item asking participants about the comprehensibility of the instructions in the app on a four-point Likert scale from 1 (*not at all*) to 4 (*very*). The UEQ-S consists of eight pairs of terms related to user experience with opposite meanings (e.g., complicated – easy) that are rated on a seven-point Likert scale from − 3 (*fully agree with negative term*) to + 3 (*fully agree with positive term*). The UEQ-S has two subscales (pragmatic quality and hedonic quality), and an overall indicator of user experience can be computed.

*Acceptability* of the training is operationalized with the ‘hedonic quality’ scale of the UEQ-S and 12 additional self-generated items, all rated on a four-point Likert scale from 1 (*not at all*) to 4 (*very*), and four open questions will be directed toward participants to gain feedback and suggestions for app improvement (see Supplementary Table 3 for the whole measure). Additionally, eight open-ended questions will be asked by the study therapist after the first and after the second training phases.

#### Kinesthesia measures

For the analysis of (affective) facial expression, videos will be recorded using the built-in smartphone camera, which will be positioned in front of the participant at a distance of approximately 40 cm. A video for each emotion induction and training trial will be recorded, adding up to a total of 44 videos. In addition to these smartphone videos, two high-resolution RGB cameras (Sony SRG-300 H) will be used, one of them positioned directly in front of and the other to one side of the participant, both approximately 1.2 m away. Together, the cameras will record the participant’s face and upper body. We will extract information on facial expression (i.e., facial action units, eye gaze, head position, and facial emotions) from recordings of the front camera and information on body posture from the side camera. A third RGB camera will focus on the study therapist during the diagnostic session and training for adherence checks. Additionally, we will use an Azure Kinect depth camera (Microsoft, Redmond, WA, USA) to estimate participants’ body posture using the Azure Body Tracking SDK.

#### Further psychophysiological measures

EMG (bipolar) of the M. corrugator supercilii, M. zygomaticus major, M. orbicularis oculi, and M. masseter (the latter only in the AFE condition) will be assessed with the Biopac MP160 (Biopac Inc. Goleta, CA, USA) system. EMG measures will be assessed in a subsample (100% of the participants in the AFE condition and randomly chosen 50% of the participants in the other three conditions) to reduce participant burden. To assess kinesthesia markers potentially relevant to depression, also sensor-based physiological data will be recorded. We will use the Biopac MP160 system to record electrocardiogram (ECG) and respiration signals, from which we will extract heart rate (variability) (bipolar) and respiration rate (variability). In addition, we will use a novel, non-invasive radar-based technology positioned approximately 0.5 m behind participants to assess respiration and heart rate (variability). Here, for cross-validation purposes, we will employ two radar systems, a 24 GHz and a 61 GHz system. The radar-based technology has been shown to be a valid contactless measure of respiration and heart rate [60].

#### App data

In the CR and CR + AFE conditions, the investigator will rate the credibility of participant’s display of emotions in their voice, facial expression, and body posture on a Likert scale from 0 to 5 via the EmpkinS-EKSpression app. The app will record timestamps for each action during the training. Moreover, it will record which statements participants selected to express approval/rejection, affirmation, and self-support in the CR and CR + AFE conditions.

### Statistical analysis plan

Analyses will be conducted via SPSS Version 29 for Windows [61] and R Studio [37]. While analyses will focus on a per-protocol approach, intention-to-treat-analyses using imputations for missing data will also be conducted. In accordance with the CONSORT statement [36], number and reasons for dropout will be recorded and reported for each condition.

#### Preprocessing of kinesthesia and further psychophysiological data

Video data will be primarily processed with the FaceReader software [62], which offers an extensive array of visual features, including probabilities associated with basic emotions such as neutral, happy, sad, angry, surprised, scared, disgusted, and contempt - all discernible within each video frame. Additionally, it provides measurements of valence and arousal that are essential for understanding emotional states. Furthermore, it allows the extraction of so-called Facial Action Units (e.g., “Eyes Closed”, “Lip Stretcher”), offering insights into the intensity of expressions ranging between 0 and 1, and estimates head position and orientation in 3D (e.g., pitch, yaw, and roll rotation angles), complemented by 2D and 3D facial landmarks. The Facial Action Units and their variations provide detailed information on facial expressions and movements [63]. With regard to videos recorded with the Azure Kinect depth camera, we will extract depth information of body posture (i.e., the distance of each body part from the camera). Psychophysiological data, i.e., EMG, ECG, and respiration signals, will be preprocessed by extracting generic as well as specific features using various methods, particularly focusing on bio-signal feature extraction. Generic features of all psychophysiological data include mean, standard deviation, minimum, and maximum. Specific features of EMG signals include mean absolute value (MAV) and root mean square (RMS), specific features of ECG signals include heart rate and RR intervals, and specific features of respiration signals include respiration rate and respiration rate variability.

Cross-sectional comparisons between diagnostic groups (i.e., with vs. without depression) regarding baseline demographic, health-related, and clinical characteristics will be conducted using *t*-tests for continuous and Χ^2^-contingency tables for dichotomous variables, respectively.

#### Efficacy of the reappraisal training augmented with facial expressions

In order to study the effectiveness of the EmpkinS-EKSpression app-based interventions, generalized linear models (GLMs) of the between-subject factors “diagnostic group” (with depression vs. without depression) and “intervention condition” (CR vs. CR + AFE vs. AFE vs. sham control), as well as the within-subject factor “time of assessment” will be applied in a stepwise fashion.

Regarding the primary outcome of depressed mood, we will first examine whether the mood induction phase *induces* depressed mood using a paired *t*-test with the pre- and post-mood-ratings as dependent variable (manipulation check). Further, hierarchical linear models (HLMs) will be implemented to assess whether the EmpkinS-EKSpression app-based interventions are effective in *reducing* depressed mood. As a first step, we will compare the CR condition with the sham control condition and the CR + AFE with the CR condition to study whether cognitive reappraisal training can be augmented with anti-depressive kinesthesia. As a second step, we will compare the AFE condition with the sham control condition. For continuously assessed secondary outcomes (i.e., positive mood, app data), corresponding analyses will be conducted.

With regard to secondary outcomes (i.e., GRID-HAMD, CES-D, current suicidality, ERSQ-ES, DAS-A, ATQ-R) assessed at pre-treatment, post-treatment, and/or follow-up, as well as pre/post mood-ratings, we will implement mixed analyses of variance (ANOVAs), again with the between-subject-factor, “study condition” (CR, CR + AFE, AFE, sham control) and the within-subject factor, “time.” Feasibility measures (treatment integrity, compliance, usability, acceptability) will be descriptively analyzed and contrasted between study conditions utilizing one-way ANOVAs. Finally, changes in kinesthesia and other psychophysiological measures over the course of the intervention, as well as differences between diagnostic groups and intervention conditions will be examined using, again, HLMs. All analyses will be conducted using the whole sample, as well as with the added between-subject-factor, “diagnostic group” (with vs. without depression). All analyses will be conducted with a significance level of α = 0.05. One-tailed tests will be used when the algebraic sign of the test statistic is specified; otherwise, two-tailed tests will be used.

#### Automated detection of depression

Machine learning methods will be implemented to examine the automated discrimination between individuals with and without depression based on kinesthesia and other psychophysiological data (i.e., facial expression and body posture from video data, facial muscle activity from EMG, respiration, heart rate (variability), and verbal acoustics from audio recordings). Initially, only video data (facial expression/body posture) will be used. Subsequently, psychophysiological data and verbal acoustics data will supplement the video dataset, providing additional modalities for analysis. Ultimately, our long-term goal is to adopt a multi-modal machine learning approach to leverage all data modalities available to enhance the accuracy and robustness of depression identification.

For the classification task (i.e., discrimination between individuals with and without depression), different machine learning pipelines will be trained. This will consist of different steps, from pre-processing and normalization to feature selection and actual classification. After the machine learning process, we will use explainable artificial intelligence techniques to identify discriminative features and to interpret the output of the machine learning models. To split the data into training and test sets, we will employ a standard train-test split using the scikit-lear library train_test_split function with a fixed random seed and allocating 80% of the dataset for model training and reserving 20% for evaluation. This approach ensures reliable performance estimation while guarding against overfitting. The performance of the classification models will be evaluated using established metrics such as sensitivity (i.e., $\:\frac{number\:of\:true\:positives}{number\:of\:true\:positives+number\:of\:false\:negatives})$ and specificity (i.e., $\:\frac{number\:of\:true\:negatives}{number\:of\:true\:negatives+number\:of\:false\:positives}$), which are indicative of the classification task’s performance with a benchmark of 80%. Furthermore, we will test whether the balanced accuracy (i.e., $\:\frac{sensitivity\:+\:specificity}{2}$) will exceed the null distribution.

Additionally, we will predict depressive symptom severity using machine learning-based regression models. The training of the models for the regression task will follow the same steps as for the classification task. As error metrics for the correspondence between clinical and machine-learning data, we will use Mean Absolute Error (MAE) and Root Mean Squared Error (RMSE). We will evaluate different machine learning models for both classification and regression tasks, such as k-Nearest Neighbour, Support Vector Machines, Decision Trees, Random Forests, and Artificial Neural Networks, to select the best-performing model based on predefined error metrics.

## Discussion

The aim of this study will be to evaluate the extent to which cognitive reappraisal in depression can be enhanced by deliberately performing anti-depressive facial expressions. Building on the ICS Theory of depression [12] and previous studies suggesting direct effects of manipulating kinesthesia (particularly facial expressions), on depressed mood [27, 28, 35], we assume that performing anti-depressive facial expressions during a cognitive reappraisal task will facilitate the invalidation of depressogenic cognitions and, hence, encourage a change in depressed mood. Should our hypothesis be confirmed, our findings would provide further evidence for the importance of the interaction of cognitive and implicational processes for the genesis of (sometimes clinically significant) affective states. Regarding clinical practice, findings from the present study could be used to further improve cognitive interventions for depression. Moreover, if the EmpkinS-EKSpression training demonstrates significant effects, it could be employed as a standalone or adjunct intervention for depression. Certainly, a more automated version of the app would greatly facilitate its dissemination, and so we will develop machine learning algorithms that enable automated facial expression recognition when these need to be coded before providing patients critical feedback. In the long term, our aim is to develop a fully-automated biofeedback training that outpatients can utilize solely with the aid of their smartphones, and to design the training flexibly enough that it can enhance in- or outpatient CBT, bridge treatment waiting times, and/or prevent relapses after successful CBT.

Further, the assessment of objective indicators of kinesthesia and the general body state of participants with and without depression (i.e., video data, psychophysiological measures, voice recordings), and the detection of patterns associated with depression using these indicators together with modern machine learning methods may have significant diagnostic implications. Automated detection of depression would open new ways to diagnose and monitor depression in a way that would not interfere with patients’ activities, and would be freed of biases that typically compromise the validity of self-report instruments [64].

Major limitations of the study include the short intervention period. It is unlikely that we will achieve long-term effects on depressed mood or effects on overall depressive symptom severity with a single intervention. Instead, the current study can serve as a proof of principle. If beneficial effects on depressed mood are found, future research should further develop the intervention (e.g., by introducing extended intervention periods) to achieve more sustained effects on depressive symptoms. Second, the planned time-consuming setup and ongoing collection of physiological data proposed here might distract participants from the intervention itself. Third, because the training is carried out in a highly standardized laboratory setting, this will limit the ecological validity of our results. Future studies should use an automated version of the training to investigate its efficacy in ambulatory settings. Fourth, we currently use standardized sets of stimulus statements, unlikely to be equally relevant for all participants. Fifth, facial EMG sensors and their cables might disturb the encoding of visual features in facial expression video data. Therefore, we are currently exploring ways of assessing EMG with wireless sensors and innovative, laser-based methods.

## Trial status

Recruitment for the RCT started in January 2023 and is currently ongoing. It is expected to be completed in January 2025.

## Electronic supplementary material

Below is the link to the electronic supplementary material.

Supplementary Material 1

## Data Availability

No datasets were generated or analysed during the current study.

## Declarations

Participants will provide written informed consent: (1) via Unipark for the screening and (2) before the initial diagnostic session for the rest of the study. In the case of study inclusion, each participant will receive a personal code. The password-protected list connecting the code with personal information will be stored separately from study data accessible by study staff only.

## Abbreviations

AFE: Anti-depressive facial expressions
ANOVA: Analysis of variance
ATQ-R: Automatic Thoughts Questionnaire-Revised
CBT: Cognitive behavioural therapy
CES-D: Centre for Epidemiological Studies Depression Scale
CR: Cognitive reappraisal
CR + AFE: Cognitive reappraisal enhanced by instructions to display anti-depressive facial expressions
CRC: Collaborative Research Center
DAS-A: Dysfunctional Attitude Scale version A
ECG: Electrocardiogram
EMG: Electromyography
ERSQ-ES A: Part A of the Self-Report Instrument for the Assessment of Emotion-Specific Regulation Skills
GLM: Generalized linear model
HAMD: Hamilton Rating Scale for Depression
HLM: Hierarchical linear model
ICS: Interacting Cognitive Subsystems Theory
MAE: Mean absolute error
MAV: Mean absolute value
PHQ: Patient Health Questionnaire
RCT: Randomized controlled trial
RMS: Root mean square
RMSE: Root mean square error
SCID-5-CV: Structured Clinical Interview for DSM-5 Disorders – Clinician version
UEQ-S: User Experience Questionnaire, short version
