# Supplementary material for: Facing depression: evaluating the efficacy of the EmpkinS-EKSpression reappraisal training augmented with facial expressions – protocol of a randomized controlled trial
Source: BMC Psychiatry. 2024 Dec 12;24:896. doi: 10.1186/s12888-024-06361-3 (PMC11636037; doi:10.1186/s12888-024-06361-3)
Supplement: Supplementary file 1 — Supplementary Material 1 [file 12888_2024_6361_MOESM1_ESM.docx]

Supplementary Material

# Supplementary Table 1

*Training in the AFE-Condition*

| **Trials** | **Instruction** | **Target muscles** |
| --- | --- | --- |
| **Relaxation** | | |
| 1-3 and 21-23 | relax the forehead | m. corrugartor supercilii |
| 4-6 and 24-26 | relax the jaw | m. masseter |
| 7-9 and 27-29 | relax the whole face (forehead and jaw) | m. corrugator supercilii & m. masseter |
| **Activation** | | |
| 10-12 and 30-32 | smile with the mouth | m. zygomaticus |
| 13-15 and 33-35 | smile with the mouth and eyes | m. zygomaticus & m. orbicularis oculi |
| 16-18 and 36-38 | smile all over the face | m. zygomaticus & m. orbicularis oculi |
| **Combination** | | |
| 19-20 and 39-40 | first relax the whole face, then smile all over the face | m. corrugator supercilii & m. masseter & m. zygomaticus & m. orbicularis oculi |

*Note*. AFE = facial muscle training focusing on anti-depressive facial expressions.

**Supplementary Table 2**

*Item Order of the Combined SCID-5-CV and GRID-HAMD*

| Item no. | SCID/HAMD-Item | Symptom |
| --- | --- | --- |
| 1 | A1 | Depressed mood |
| 2 | A2 | Diminished interest or pleasure |
| 3 | HAMD 1 | Depressed mood |
| 4 | HAMD 18 | Diurnal variation |
| 5 | HAMD 7 | Work and activities |
| 6 | HAMD 22 | Helplessness |
| 7 | HAMD 23 | Hopelessness |
| 8 | A3 | Weight loss/gain or decrease/increase in appetite |
| 9 | HAMD 12 | Loss of appetite (somatic symptoms, gastrointestinal) |
| 10 | HAMD 16 | Loss of weight |
| 11 | A4 | Insomnia/hypersomnia |
| 12 | HAMD 4 | Insomnia early |
| 13 | HAMD 5 | Insomnia middle |
| 14 | HAMD 6 | Insomnia late |
| 15 | A5 | Psychomotor agitation or retardation |
| 16 | HAMD 8 | Psychomotor retardation |
| 17 | HAMD 9 | Psychomotor agitation |
| 18 | A6 | Fatigue/loss of energy |
| 19 | HAMD 13 | Somatic symptoms, general |
| 20 | HAMD 14 | Sexual interest (genital symptoms) |
| 21 | A7 | Feelings of worthlessness or excessive/inappropriate guilt |
| 22 | HAMD 2 | Guilt |
| 23 | HAMD 24 | Worthlessness |
| 24 | A8 | Diminished ability to think/concentrate, or indecisiveness |
| 25 | HAMD 10 | Anxiety, psychological |
| 26 | HAMD 11 | Anxiety, somatic |
| 27 | HAMD 15 | Hypochondriasis |
| 28 | HAMD 19 | Depersonalization/derealization |
| 29 | HAMD 20 | Paranoid symptoms |
| 30 | HAMD 21 | Obsessional/compulsive symptoms |
| 31 | A9 | Suicidality |
| 32 | HAMD 3 | Suicide |
| 33 | A10 |  |
| 34 | HAMD 17 | Insight |

*Note*. Items starting with “A” are the items of the SCID-5-CV and describe the Symptoms of major depression according to the Diagnostic and statistical manual of mental disorders (American Psychiatric Association, 2013). SCID-5-CV = Structured Clinical Interview for DSM-5 Disorders - Clinician version; HAMD = Hamilton Rating Scale for Depression.

**Supplementary Table 3**

*Self-Developed Items to Assess Usability and Acceptability*

How understandable did you find the instructions in the app?

| Not at all | A little | Fairly | Very |
| --- | --- | --- | --- |
| 🔿 | 🔿 | 🔿 | 🔿 |

How helpful did you find…

|  | Not at all | A little | Fairly | Very |
| --- | --- | --- | --- | --- |
| …the first part of the training | 🔿 | 🔿 | 🔿 | 🔿 |
| …the second part of the training | 🔿 | 🔿 | 🔿 | 🔿 |
| …the training as a whole | 🔿 | 🔿 | 🔿 | 🔿 |

Did you enjoy the training?

| 🔿 | Not at all |
| --- | --- |
| 🔿 | A little |
| 🔿 | Fairly |
| 🔿 | Very |

How likely would you continue the training regularly (e.g., 1x/week) if you had the opportunity to do so?

| 🔿 | Not at all |
| --- | --- |
| 🔿 | A little |
| 🔿 | Fairly |
| 🔿 | Very |

Has the training helped you to change your negative thoughts?

| 🔿 | Not at all |
| --- | --- |
| 🔿 | A little |
| 🔿 | Fairly |
| 🔿 | Very |

Has the training helped you to cope with your negative thoughts more appropriately?

| 🔿 | Not at all |
| --- | --- |
| 🔿 | A little |
| 🔿 | Fairly |
| 🔿 | Very |

How strenuous did you find the training?

| 🔿 | Not at all |
| --- | --- |
| 🔿 | A little |
| 🔿 | Fairly |
| 🔿 | Very |

Did you find the time required for the training appropriate?

| 🔿 | Far too high |
| --- | --- |
| 🔿 | A little too high |
| 🔿 | Appropriate |
| 🔿 | A little too short |
| 🔿 | Far too short |

Did you find it difficult to concentrate on the training?

| 🔿 | Not at all |
| --- | --- |
| 🔿 | A little |
| 🔿 | Fairly |
| 🔿 | Very |

How stressful did you find participating in the study?

| 🔿 | Not at all |
| --- | --- |
| 🔿 | A little |
| 🔿 | Fairly |
| 🔿 | Very |

How satisfied were you with the training in general?

| 🔿 | Not at all |
| --- | --- |
| 🔿 | A little |
| 🔿 | Fairly |
| 🔿 | Very |

Why did you take part in the study?

| 🔿 | Financial incentive |
| --- | --- |
| 🔿 | Pastime |
| 🔿 | Interest |
| 🔿 | Hoping for positive effects of the intervention |
| 🔿 | Other: ________________________ |

Would you recommend the training to friends or acquaintances?

| 🔿 | Yes |
| --- | --- |
| 🔿 | No |

What did you like less?

What would you change/add in the training?

What did you like most about the training?

What do you take away from the training?
